# The effects of the DNA methyltranfserases inhibitor 5‐Azacitidine on ageing, oxidative stress and DNA methylation of adipose derived stem cells

**DOI:** 10.1111/jcmm.12972

**Published:** 2016-12-20

**Authors:** Katarzyna Kornicka, Krzysztof Marycz, Monika Marędziak, Krzysztof A. Tomaszewski, Jakub Nicpoń

**Affiliations:** ^1^ Faculty of BiologyUniversity of Environmental and Life SciencesWrocławPoland; ^2^ Wroclaw Research Centre EIT+WrocławPoland; ^3^ Faculty of Veterinary MedicineUniversity of Environmental and Life SciencesWrocławPoland; ^4^ Department of AnatomyJagiellonian University Medical CollegeKrakowPoland; ^5^ Department of SurgeryFaculty of Veterinary MedicineUniversity of Environmental and Life Sciences WroclawWroclawPoland

**Keywords:** mesenchymal stem cells, ageing, methylation, oxidative stress, 5‐Azacitidine

## Abstract

Human adipose tissue is a great source of adult mesenchymal stem cells (MSCs) which are recognized from their ability to self‐renew and differentiation into multiple lineages. MSCs have promised a vast therapeutic potential in treatment many diseases including tissue injury and immune disorders. However, their regenerative potential profoundly depends on patients’ age. Age‐related deterioration of MSC is associated with cellular senescence mainly caused by increased DNA methylation status, accumulation of oxidative stress factors and mitochondria dysfunction. We found that DNA methyltransferase (DNMT) inhibitor i.e. 5‐Azacytidine (5‐AZA) reversed the aged phenotype of MSCs. Proliferation rate of cells cultured with 5‐AZA was increased while the accumulation of oxidative stress factors and DNA methylation status were decreased. Simultaneously the mRNA levels of TET proteins involved in demethylation process were elevated in those cells. Moreover, cells treated with 5‐AZA displayed reduced reactive oxygen species (ROS) accumulation, ameliorated superoxide dismutase activity and increased BCL‐2/BAX ratio in comparison to control group. Our results indicates that, treating MSCs with 5‐AZA can be justified therapeutic intervention, that can slow‐down and even reverse aged‐ related degenerative changes in those cells.

## Introduction

The populations of many regions, especially in developed countries, are rapidly growing older. 1. That fact become a real challenge for national healthcare systems, economy and medicine. Although the portion of the elderly that faces severe disability is decreasing, they still share a great susceptibility to various chronic conditions and disabilities like osteoporosis and osteoarthritis. Thus, elderly patients, become a group that require an effective and rapid therapeutic treatment. Recently, a lot of attention has been paid to regenerative medicine‐ a novel therapeutic strategy that include preparation of functionalized biomaterials, development of sufficient pharmaceutical agents based on cellular therapy 2, 3.

Regenerative medicine and/or tissue engineering has a great therapeutic potential and might be an effective strategy for treating elderly patients. Currently, there are 344 registered clinical trials in different phases aimed at evaluating the potential of mesenchymal stem cells (MSC) based therapy worldwide 4, 5. Due to their accessibility and convenient expansion protocols, MSC have been recognized as promising candidates for cellular therapy. The potential of MSC therapy involves their unique characteristics: self‐renewing and multilineage potential to differentiate into cell types of mesodermal origin. Moreover, MSC can migrate to the inflammation site and exert potent immunosuppressive and anti‐inflammatory effects through interactions between lymphocytes. Because of their characteristics, MSC are being explored in trials of various conditions, including tissue injury and immune disorders 6, 7, 8, 9. What is worth noting, many independent clinical trials in regenerative medicine, already showed the effectiveness of MSCs engraftment in regeneration of musculoskeletal system 10 neuronal 11, 12 and cardiac tissue 13, 14. However, as recently showed MSCs regenerative potential decreases with patients age. The impairment of MSCs properties, including decreased proliferation rate, increased apoptosis and senescence, might lead to unsatisfactory clinical results 15, 16. Hence, the analysis of MSCs molecular condition on various levels, before their application, seems to be highly reasonable. Especially, while taking into consideration the ageing phenotypes of those cells caused by different intracellular factors and also by the ageing of stem cells niche itself.

Several studies, including ours, showed that MSCs isolated from adipose tissue (hASCs) of elderly patient are characterized by the elevated oxidative stress levels, and therefore suffer from increased senescence, impairment of cellular viability and activity 15, 17, 18. The both: oxidative stress and epigenetic modifications of the genome are recognized as crucial factors initiating MSCs ageing and senescence, that in consequence leads to the impairment of their regenerative potential 19, 20. Increased accumulation of reactive oxygen species (ROS) and nitric oxide, simultaneously with decreased anti‐oxidative protection coming from superoxide dismutase (SOD) activity leads to the state of permanent growth arrest or apoptosis 21. In this case the initiation of apoptosis is carried out by up‐regulation of p21, p53 (tumour suppressor), BAX expression, cytochrome C relocation, chromatin condensation and remodelling 22, 23. Moreover, changes in epigenetic status of MSCs including DNA and histone methylation can affect the MSCs viability and proliferative activity: both crucial in the context of cellular therapies 24. Efforts have been made to applied histone deacetylase inhibitors (HDAC) as an anti‐ageing factor in MSCs culture. Even if HDAC has showed to improve osteogenic differentiation process of those cells, they simultaneously slowed down the viability through DNA damage and cell‐cycle inhibition 25, 26. It also has been proved, that DNA demethylation can be induced by the application of DNA methyltransferase (DNMT) inhibitors i.e. 5‐Azacitidine (5‐AZA) 27. That agent has been demonstrated as an effective factor that inhibits cancer cells viability, and thus was proposed as a therapeutic agent for treating an acute myelogenous leukaemia 28, 29. It was also observed that 5‐AZA is easily incorporated into DNA and inhibits methylation pattern of specific gene regions, simultaneously with activation of associated genes 30. Epigenetic changes induced by 5‐AZA in hepatocyte‐like cells lead to significantly improved metabolic and enzymatic activities compared to non‐treated cells 31. These findings prompted the other research groups to test if 5‐AZA, because of their demethylation ability, might affect the MSCs viability and have found it's beneficial effect in the course of osteogenic differentiation process. Just recently, the explanation of DNA demethylation mechanism, however in embryonic stem cells (ESC) has been proposed. DNA‐demethylation was observed by induction of TET gene expression, that initiate the conversion of nuclear 5‐methylcyt‐osine (5 mC) to 5‐hydroxymethylcytosine (5 hmC). These in consequence leads to improvement of the self‐renewal ability of ESC and maintain their pluripotent status 32.

Although MSCs for cell therapy have been shown to be safe and effective, there are still challenges that need to be tackled before their wide application in the clinical practice. Especially the impaired proliferative activity caused by senescence and decreased ‘stemness’ status of those cells needs to be overcome. One of the solutions is treating MSCs *in vitro* with different agents that may lead to their rejuvenation and finally to effective and successful therapy, especially when using cells from elderly donors which suffer from age‐related deterioration.

This study investigated the effects of 5‐Azacitidine on the viability and proliferative activity of ASCs of healthy, however elderly donors. Moreover, we analysed correlation between ROS/nitric oxide *versus* SOD activity, hASCs growth kinetics, apoptotic *versus* anti‐apoptotic genes expression and mitochondrial morphological imperfections. Finally, bearing in mind the fact, that 5‐Azacitidine was reported as a demethylation agents in embryonic stem cells, that maintain their pluripotency, we tested the expression level of TET2/3 genes in relation to 5‐methylocysteine conversion for 5‐hydroxymethylcysteine.

## Materials and methods

All reagents used in this experiment were purchased from Sigma‐Aldrich (Poznan, Poland), unless indicated otherwise.

All experimental procedures were approved by the II Local Ethics Committee of Environmental and Life Sciences University (Chelmonskiego 38C, 51‐630 Wroclaw, Poland; decision No. 84/2012). An informed, written consent for using the samples for research purposes was obtained from all patients prior to surgery. The study has been performed in accordance with the ethical standards laid down in the 1964 Declaration of Helsinki and its later amendments.

### Isolation of adipose‐derived mesenchymal stem cells (ASCs)

Human subcutaneous adipose tissue was collected from both male and female subjects; age range 52–78 (median age equalled 67). After surgical harvesting adipose tissue samples were placed in Hank's Balanced Salt Solution (HBSS) and processed under the same, sterile conditions. Isolation of ASCs was performed in accordance to a previously described protocol 33. Briefly, tissue fragments were washed extensively with HBSS supplemented with 1% antibiotic‐antimycotic solution (penicillin/streptomycin/amphotericin B) and minced. The extracellular matrix was digested with collagenase type I (1 mg/ml) for 40 min. at 37°C and 5% CO_2_. Next, tissue homogenates were centrifuged for 10 min. at 1200 × g. The supernatants were discarded and the pellets of stromal vascular fraction (SVF) containing ASCs were washed with HBSS and centrifuged again for 4 min. at 300 × g. The supernatant was discarded and the pellet was re‐suspended in the culture medium. The cell suspension was then transferred to a culture flask. Primary culture of ASCs’ was designated as ‘passage 0’. To prepare cells for experiment, they were passaged three times.

### Flow cytometer analysis

Human ASCs were recognized by immunophenotyping using fluorochrome conjugated monoclonal antibodies specific for: CD29, CD34, CD45, CD90, CD73b and CD44. isotype‐matched antibodies were used as controls. Due to immunophenotyping ASCs were detached using TrypLE^™^ Express solution, washed with HBSS contained 2% FBS and re‐suspended at total of 5*10^5^ cells/ml. Cell suspension was incubated at 4°C for 20 min. with the specific antibodies pre‐conjugated with allophycocyanin (APC), peridinin chlorphyllprotein (PerCP), fluorescein isothiocyanate (FITC) or phycoerythrin (PE). At least ten thousand stained cells were acquired and analysed by Becton Dickinson FACS Calibur flow cytometer. The samples were analysed using CellQuest Pro software (Becton Dickinson, Franklin Lanes, New Jersey, USA).

### Multipotency assay of ASCs

Osteogenic, chondrogenic and adipogenic differentiation of cells were induced using commercial kits (STEMPRO^®^ Osteogenesis Differentiation Kit and STEMPRO^®^ Adipogenesis Differentiation Kit, both Life Technologies, Waltham, Massachusetts, USA) in accordance to manufacturers’ protocols. In order to perform the test, the cells were seeded in a 24‐well plate at the initial density of 2 × 10^4^ and the media were changed every 2 days. Experiments were carried out simultaneously, each in triplicate. Stimulation of osteo‐ and chondrogenesis lasted 21 days, while stimulation towards adipogenic lineage lasted for 14 days. Cultures expanded in standard growth medium were used as a control to allow for establishing differentiation effectiveness. Multi‐lineage differentiation was confirmed at 2 weeks post‐induction by cells staining. To evaluate the results of differentiation process cells were fixed with 4% ice‐cold paraformaldehyde (PFA) and specific stainings were performed. Extracellular mineralized matrix was visualized with Alizarin Red dye, while the formation of proteoglycans was confirmed by Safranin O. Intracellular lipid droplets were stained red with Oil Red O. Cells were observed under an inverted microscope (AxioObserverA1, Zeiss (Oberkochen, Germany)) and photographs were acquired using Cannon PowerShot digital camera.

### Cell culture

The cells were cultured in DMEM with the F‐12 Ham's nutrient supplemented with 10% of foetal bovine serum (FBS) and 1% P/S/A solution. During the experiment, the cells were cultured under aseptic and constant conditions in an incubator (37°C, 5% CO_2_ and 95% humidity). The media were changed every 2 days and the cells were passaged using trypsin solution (TrypLE^™^ Express; Life Technologies, Warsaw, Poland) according to manufacturers’ instructions after reaching 80% confluence.

### Pre‐treatment of ASCs with 5‐AZA

Prior experiment ASCs were seeded onto 24‐well plates at the density of 20 × 10^3^ per well. After cell attachment culture medium was replaced with medium containing 5‐AZA (1 μM). After total pre‐treatment (48 h) with 5‐AZA, cells were maintained in standard culture medium again. Cells cultured in standard medium (DMEM, 10% of FBS, 1% P/S/A) without addition of 5‐AZA served as a control group. Non‐treated cells served as a control for comparison with the test culture. All experiments were performed after seventh day of experiment.

### Cell viability, population doubling time and colony forming unit‐fibroblasts (CFU‐fs) assay

Cell proliferation rate was evaluated using 10% resazurin‐based dye‐TOX‐8 (Sigma‐Aldrich) following manufacturer's protocols. To perform the test, culture media were removed and replaced with culture medium containing 10% of the dye. The cells were then incubated with the dye in the CO_2_ incubator, 37°C for 2 hrs. Supernatants were collected and transferred to 96‐well plate to perform the spectrophotometric assay (BMG Labtech, Ortenberg, Germany). The absorbance of the supernatants was measured at a wavelength of 600 nm for resazurin, and 690 nm reference wavelength. Each measurement included a blank sample, containing medium without cells. The number of cells was estimated on the basis of standard curve, generated during the experiment. To prepare the curve, cells were seeded at the density of 20 × 10^3^, 40 × 10^3^, 80 × 10^3^ per well and dye absorbance was measured in relation to certain cells number. Linear trendline equation allowed estimating cells number throughout the experiment.

Population doubling time (PDT) was assessed with the support of a population doubling time on‐line calculator (http://www.doubling-time.com/compute.php) using formula presented below. Initial concentration equalled 2 × 10^4^ cells, (initial seeding density) while final concentration equalled the number of cells in culture on the seventh day of the experiment.

$$\text{PDT}=\frac{\text{duration}*log(2)}{log(\text{Final Concentration})-log(\text{Initial Concentation})}$$

To evaluate the ability of cells to form colonies, ASCs were seeded in six‐well plates at a density of 100 cells per well and inoculated into a culture medium. The medium was changed every 2 days and cultures were maintained for 7 days. After fixation in 4% ice‐cold paraformaldehyde, cells were stained with pararosaniline solution and colonies of more than 50 cells were counted and documented by a Power Shot Camera. The efficiency of colony forming was calculated using the formula presented below, as described elsewhere 34.

$$\text{CFU‐fs}\left[\%\right]=\frac{\text{the number of colonies}}{\text{initial cell number}}\times 100\%$$

### Measurement of DNA synthesis: BrdU assay

DNA synthesis was assessed by measuring the incorporation of 5‐bromo‐2‐deoxyuridine (BrdU) into cellular DNA. The test was performed after 48, 96 and 168 hrs of culture. The assay was performed with BrdU Cell Proliferation ELISA Kit (Abcam, Milton, Cambridge CB4 0FL, United Kingdom) in accordance to manufacturer's instruction. Briefly, cells were incubated with BrdU overnight at 37°C. Next, cells were fixed and DNA was denaturated. Incorporation of BrdU was determined by incubation with anti‐BrdU monoclonal antibody. Goat antimouse IgG conjugated with horseradish peroxidase (HRP), was used as a secondary antibody. Colour reaction was developed using 3,3,5,5‐tetramethylbenzidine (TMB). Signal intensity was measured with a spectrophotometer plate reader (BMG Labtech, Ortenberg, Germany), at a wavelength of 450/550 nm.

### Examination of ASCs morphology and ultrastructure

Cell morphology was assessed using an inverted epifluorescent microscope (Zeiss, Axio Observer A.1). The preparation of cells for fluorescent microscopy included following stages (i) washing three times with HBSS, (ii) fixation in 4% PFA, (iii) washing with HBSS, (iv) permeabilization by 0.2% Tween 20 in HBSS for 15 min. at room temperature, (v) washing with HBSS. Next, actin filaments were stained using atto‐565‐labeled phalloidin at dilution 1:800 with HBSS for 40 min. in the dark at room temperature and cells’ nuclei were counterstained with diamidino‐2‐phenylindole (DAPI; 1:1000) for 5 min. In addition, percentage of enlarged cells was calculated based on the representative images (cells were qualified as ‘enlarged’ when cell diameter was greater than >280 μm) as spread ‐out, flattened morphology and increased nuclei size, typical features associated with ASC senescence.

Detailed characteristics of ASCs morphology were evaluated using scanning electron microscope (Zeiss EVO LS15). After fixation in 4% PFA cells were washed with HBSS three times and dehydrated in a graded ethanol series (from 50% to 100%). Air‐dried samples were then sputtered with gold (Scancoat Six, Crawley, United Kingdom), placed in a microscope chamber and observed using a SE1 detector, at 10 kV of filament's tension. Using SEM data, we also evaluated mean cell nuclei diameter.

For transmission electron microscopy (TEM), cells were fixed in 2.5% glutaraldehyde at 4°C. After fixation step cells were: (i) centrifuged at 2000 × g for 10 min. and rinsed with PBS for 30 min., (ii) centrifuged again, parameters as above, (iii) incubated with 1% osmium tetroxide in HBSS for 2 hrs, (iv) washed with HBSS, (v) centrifuged as described above, (vi) dehydrated in graded acetone series (30–100%), (vii) embedded using Agar Low Viscosity Resin Kit (Agar Scientific Ltd, Essex, UK). Ultrathin sections (80 nm) were collected on copper grinds. Uranyl acetate and lead citrate were used for contrasting for 30 and 15 min. respectively. The observations were carried out using Auriga60 Zeiss STEM (Oberkochen, Germany), at 20 kV filament tension.

The diameter of cell nuclei observed under SEM was calculated based on the representative images. The percentage of heterochromatin in nuclei observed under TEM was calculated by ImageJ software (Rasband, W.S., Image J, U. S. National Institutes of Health, Bethesda, Maryland, USA, http://imagej.nih.gov/ij/, 1997‐2016.).

### Visualization of tubulin, Golgi apparatus and endoplasmic reticulum (ER)

To visualize cells’ organelles commercially available kits were applied. Tubulin was stained with a red fluorescent protein, using CellLight^®^ Tubulin‐RFP, BacMam 2.0 (Life Technologies), Golgi apparatus was labelled with a green fluorescent protein, using CellLight^®^ Golgi‐GFP, BacMam 2.0 (Life Technologies), and endoplasmic reticulum was stained with a red fluorescent protein using CellFight^®^ ER‐RFP, BacMam 2.0 (Life Technologies) following manufacturers’ instructions. Briefly, cells were incubated overnight in the CO_2_ incubator, 37°C. Next cells were washed three times with HBSS, fixed in 4% PFA for 40 min. and washed again. Cells nuclei were counterstained with DAPI.

### Senescence, oxidative stress factors, ATP and DNA methylation analysis after 7 days culture

To visualize senescence‐associated β‐galactosidase, we performed staining using Senescence Cells Histochemical Staining Kit (Sigma‐Aldrich) following the manufacturer's protocol. Amount of viable and dead cells was evaluated with Cellstain Double Staining Kit (Sigma‐Aldrich). Viable cells were stained with Calcein‐AM and emitted green fluorescence, whereas dead cells’ nuclei were stained orange with Propidium Iodide. The percentage of dead cells was calculated. The cells were observed using epifluorescence microscopy and all procedures were conducted in accordance with the manufacturer's instructions. The amount of adenosine triphosphate (ATP) produced by investigated cells was performed with ATP assay kit (Abcam). All procedures were performed according to manufacturer's protocols.

To evaluate stress levels, the cells were cultured in medium without phenol red for 7 days and the tests were performed on the second and seventh day. Nitric oxide concentration was assessed with the Griess Reagent Kit (Life Technologies), SOD by SOD Assay Kit and the level of ROS was estimated with a H2DCF‐DA (Life Technologies) solution. All procedures were performed in duplicate in accordance to the manufacturer's protocols. In addition, we performed fluorescence staining for ROS using Total ROS detection kit for microscopy and flow cytometry (Enzo Life Sciences, New York, USA). To confirm whether ROS mainly come from mitochondria, we counterstained them with Mito Red dye (dilution 1:1000). All procedures were performed following manufacturers’ instructions.

Moreover, we established DNA methylation status using DNA Methylation EIA Kit (Cayman Chemical, Ann Arbor, Michigan, USA) for quantitative data. To perform the assay DNA was purified from cells using Genomic DNA Purification kit (Thermo Fisher Scientific (Waltham, Massachusetts, Stany Zjednoczone)). Next, DNA was digested using nuclease S1 (Thermo Fisher Scientific) following incubation with alkaline phosphatase at 37°C for 30 min. Samples were then boiled for 10 min. and placed on ice until use. To perform the test 400 ng of DNA was used. All procedures were conducted following manufacturers’ instructions.

### Immunofluorescence stainings

To perform the analysis, cells were fixed in 4% PFA for 40 min. and washed three times with HBSS. For permeabilization, cells were incubated with 0.5% Triton X‐100 for 20 min. at room temperature. Following HBSS wash, unspecific binding sites were blocked with blocking buffer (10% Goat Serum, 0.2% Tween‐20 in HBSS) for 45 min. Then, the cells were incubated with primary antibodies against Ki67 and Caspase‐3 (both from Abcam) diluted 1:200 in HBSS containing 1% Goat Serum and 0.2% Tween‐20. After rinsing with HBSS, cells were incubated for 1 hr with mouse anti‐rabbit secondary antibodies conjugated with atto‐594 and atto‐488 (dilution 1:200), avoiding direct light. Nuclei were counterstained by incubation with DAPI for 5 min.

To visualize 5‐methylocytosine (5‐MC) and 5‐hMC cells were: (i) fixed with 4% PFA for 40 min., (ii) permeabilized in 0.5% Triton X‐100 in HBSS, (iii) incubated with 4N HCl for 15 min., (iv) incubated with blocking buffer (10% Goat Serum, 0.2% Tween‐20 in HBSS) for 45 min. Next, cells were incubated with primary antibodies diluted 1:200 in HBSS containing 1% Goat Serum and 0.2% Tween‐20 overnight (anti‐5‐methylocytosine, ab73938, Abcam; anti‐5‐hydroxymethylocytosine,ab106918, Abcam). After washing three times with HBSS, cells were incubated with secondary antibodies conjugated with atto‐488 for 1 hr (1:200, goat anti‐rat, ab150157, Abcam; goat antimouse, ab150113, Abcam).

### Analysis of gene expression: real‐time reverse transcription polymerase chain reaction (qRT‐PCR)

On the seventh day of the experiment, cells were homogenized using 1 ml TRI Reagent. Procedure of homogenization was performed directly in the culture dish. Total RNA was isolated using the phenol–chloroform method as previously described by Chomczynski & Sacchi 35. Obtained samples were diluted in DEPC‐treated water. Both, quality and quantity of isolated total RNA were determined using nano‐spectrometer (WPA Biowave II, Hercules, California, USA). Genomic DNA digestion and cDNA synthesis were performed with PrimeScript kit (Takara, Clontech, Kusatsu, Shiga, Japan). For each reaction, 150 ng of total RNA was used. Both processes were performed in accordance with the manufacturers’ instructions using a T100 Thermal Cycler (Bio‐Rad, Hercules, California, USA).

The qRT‐PCR reactions were performed with a CFX ConnectTM Real‐Time PCR Detection System (BioRad). Reaction mixture contained 2 μl of cDNA in a total volume of 20 μl using SensiFast SYBR & Fluorescein Kit (Bioline, Cincinnati, Ohio, USA). The concentration of primers in each reaction equalled to 500 nM; primer sequences used in individual reactions are listed in Table 1. Relative gene expression analysis (Qn) was calculated in relation to the GAPDH housekeeping gene.

**Table 1:** Sequences of primers used in qPCR

Gene	Primer	Sequence 5′–3′	Amplicon length (bp)	Accesion no.
BAX	F:	ACCAAGAAGCTGAGCGAGTGTC	365	XM_011527191.1
	R:	ACAAAGATGGTCACGGTCTGCC		
BCL‐2	F:	ATCGCCCTGTGGATGACTGAG	129	NM_000633.2
	R:	CAGCCAGGAGAAATCAAACAGAGG		
TET2	F:	GAGACGCTGAGGAAATACGG	258	XM_011532044.1
	R:	TGGTGCCATAAGAGTGGACA		
TET3	F:	CAGAACGCTGTGATCGTCAT	263	XM_011532690.1
	R:	AACTTGCGAGGTGTCTTGCT		
p21	F:	GGCAGACCAGCATGACAGATTTC	72	NM_001291549
	R:	CGGATTAGGGCTTCCTCTTGG		
p53	F:	AGATAGCGATGGTCTGGC	381	NM_001126118.1
	R:	TTGGGCAGTGCTCGCTTAGT		
Lin28	F:	CCGAACCCCATGCGCACGTT	137	NM_024674.5
	R:	TTTGCAGGTGGCTGCGCCAAG		
GAPDH	F:	CATGGCCTTCCGTGTTCCTA	286	NM_017008.4
	R:	CACCACCCTGTTGCTGTAGC		

Sequences, amplicon length and accession numbers of the primer sets; BAX‐ BCL2‐Associated X Protein; BCL‐2‐ B‐cell lymphoma 2; TET2‐ tet methylcytosine dioxygenase 2; TET3‐ tet methylcytosine dioxygenase 3; p21 ‐ cyclin‐dependent kinase inhibitor 1A; p53 ‐ tumor suppressor p53; Lin28‐ lin‐28 homolog A, GAPDH ‐ glyceraldehyde 3‐phosphate dehydrogenase

Moreover, we evaluated the ratio between BCL‐2 and BAX expression in each group by dividing Qn of BCL‐2 by Qn of BAX.

### Statistical analysis

Results are shown as growth curves or box plots of at least three independent experiments, measured as triplicates or more. Statistical significance was determined using the unpaired *t*‐test or one‐way anova with Tukey's *post hoc* test (Prism5.04, GraphPad Software, CA, USA). *P* < 0.05 was considered statistically significant.

## Results

### 5‐AZA treatment maintain the expression of ASC specific surface antigens and ASC multipotency

**Figure 1:**
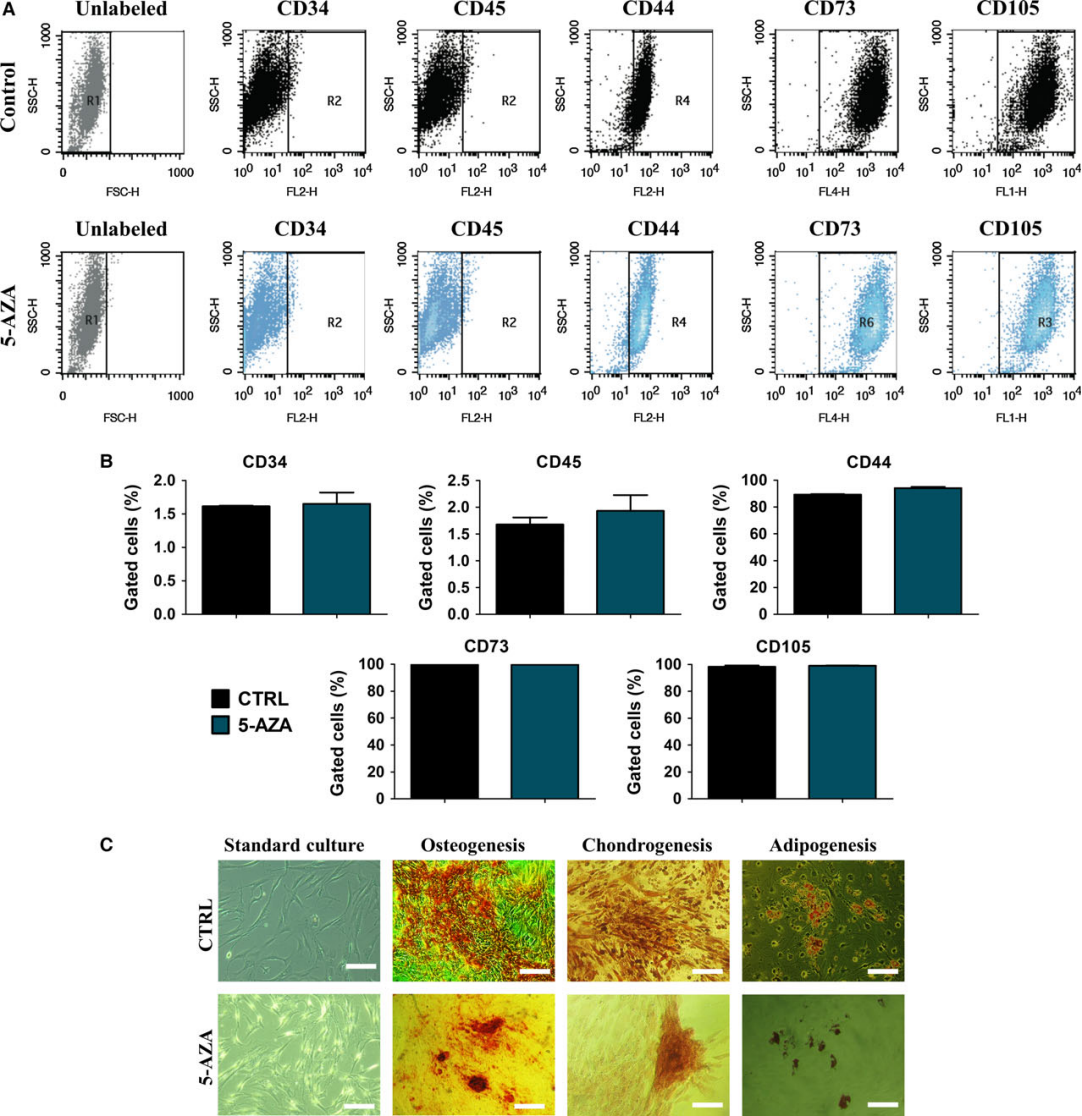
Flow cytometry and multipotency analysis of isolated hASCs. Phenotype of hASCs was determined with flow cytometry. Isolated cells were strongly positive for CD 44, CD73, CD105 surface antigens. In contrast cells lacked the expression of hematopoietic markers CD45 and CD 34 (**A,B**). Representative images from tri‐lineage assay (**C**). Osteogenesis was confirmed with Alizarin Red staining for extracellular matrix mineralization while proteoglycans formed during chondrogenesis were stained with Safranin O. Formation of intracellular lipid‐reached vacuoles as typical characteristic of adipogenic process was visualized with Oil Red O. Magnification ×100, scale bars 250 μm.

To confirm multipotent and mesenchymal character of isolated cells flow cytometry and multipotency assay were performed. Flow cytometer analysis revealed that ASCs were positive for mesenchymal markers specific for multipotent stromal stem cells, e.g. CD44, CD73 and CD105. On the contrary, ASCs showed a negative reaction with the CD34 and CD45 hematopoietic markers (Fig. 1A, B). The multipotency of isolated cells was confirmed by the positive results of osteo‐, chondro‐ and adipogenic differentiation. Cells cultured in osteogenic medium formed extracellular mineralized matrix stained with Alizarin Red. Cells cultured under chondrogenic conditions formed proteoglycan rich matrix stained with Safranin O. In adipogenic cultures, cells formed intracellular lipid vacuoles stained red with Oil Red O (Fig. 1C).

### Treatment with 5‐AZA increased ASCs proliferations as seen by increase Ki67 and decrease population doubling time

**Figure 2:**
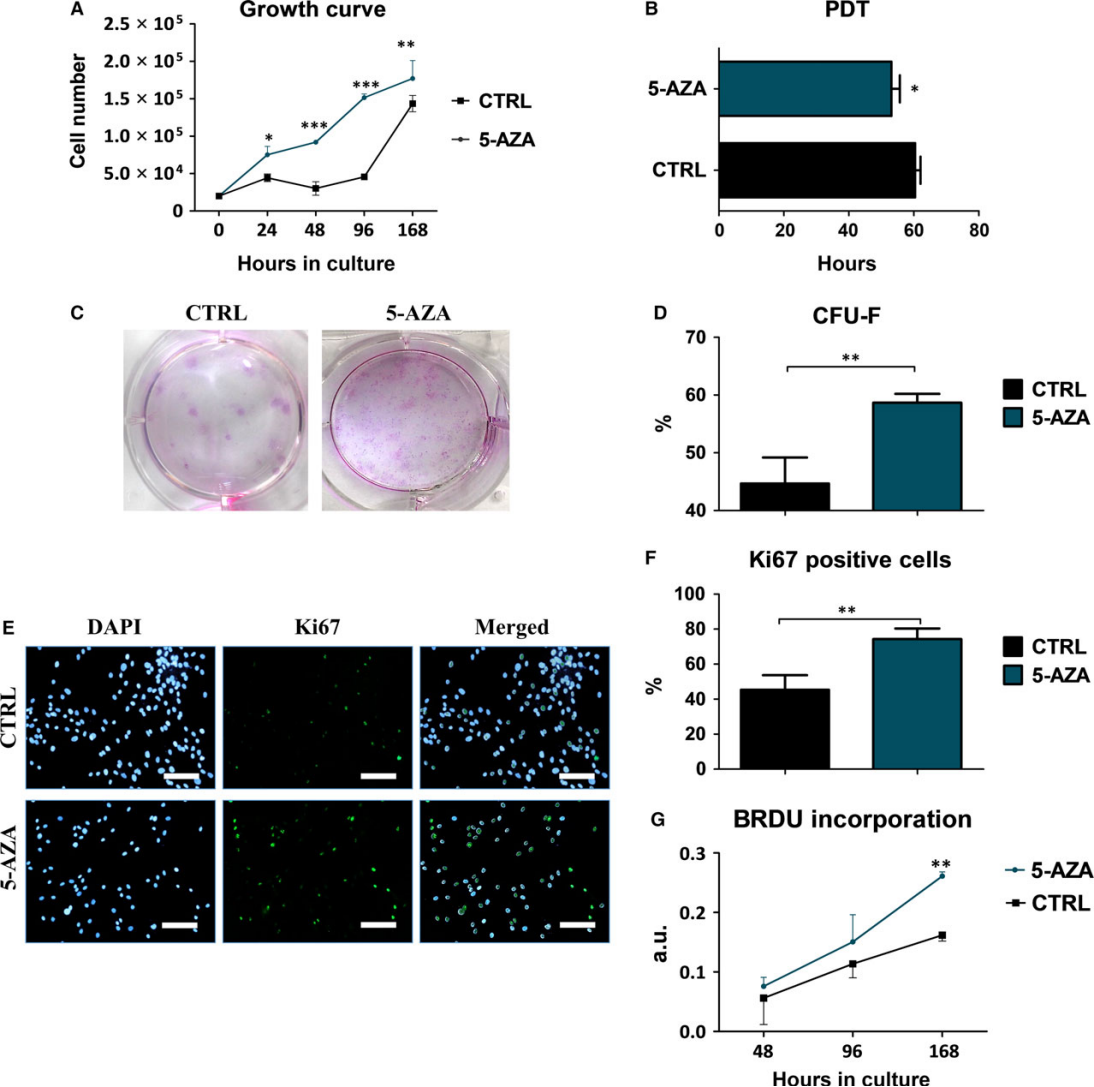
Influence of 5‐AZA treatment on hASCs growth kinetics and clonogenic potential. Mean cells number was assessed using Alamar Blue test (**A**). Cells cultured with culture medium supplemented with 5‐AZA displayed higher proliferation rate in comparison to control group during the whole experimental period. The greatest significances were observed after 48 and 96 hrs of culture (*P* < 0.001). In respect to growth curve data, mean population doubling time was calculated (PDT) (**B**). To perform the CFU assay cells were seeded at the initial density of 100 cells/per well onto six‐well culture plate. After 7 days of cultures colonies were scored and CFU‐F efficiency was calculated using mentioned equation. Obtained results showed greatest potential to form colonies in hASCs treated with 5‐AZA (*P* < 0.01). Visualization of CFU‐F colonies stained with Pararosaniline (**C**). CFU‐F assay showing percentage of colonies consisting of more than 50 cells (**D**). Proliferative cells were visualized using immunofluorescence stainings for Ki67 antigen (green), which is located in cell's nucleus during all active phases of the cell cycle (despite Go phase). Cells nuclei were counterstained with DAPI (blue) (**E**). Representative images of Ki67 were used for quantification of Ki‐67 positive cells (**F**). The obtained data points to increased proliferation of hASCs after treatment with 5‐AZA (*P* < 0.01) in comparison to control group. In addition, the relative activity of cells that were replicating their DNA was assessed with BrdU incorporation assay after 48, 96 and 168 hrs of culture (**G**). After 168 hrs of culture we noted significantly increased DNA synthesis (*P* < 0.01) in cells treated with 5‐AZA. Magnification ×100, scale bars 250 μm; *P < 0.05, **P < 0.01.

The proliferative activity of ASCs cultured both, in control and 1 μM of 5‐AZA supplemented medium was evaluated after 24, 48, 96 and 168 hrs of culture. Cell activity in control culture declined after 24 hrs and began to increase after 48 hr till the last day of the experiment. Proliferation of cells cultured with 5‐AZA was characterized by exponential cell growth during the whole experiment (Fig. 2A). Those cells displayed significant increase in number in comparison to control group on each investigated day. The greatest differences in cell number were observed after 48 and 96 hrs of culture (*P* < 0.001), while on day 7th difference was less (*P* < 0.01), probably because growth inhibition caused by high confluence on plate wells.

Moreover, time required to double the population was significantly reduced in ASCs cultured with 5‐AZA in comparison to control culture (*P* < 0.05) (Fig. 2B).

The ability of cells to form colonies originated from one cell, was established with clonogenic fibroblast precursor (CFU‐F) assay. The greatest number of colonies consisted of more than 50 cells was observed in ASCs cultured with 5‐AZA (*P* < 0.01). These data were showed qualitatively on the pictures (Fig. 2C) and quantitatively on the graph (Fig. 2D).

Proliferative activity of ASCs was also established with immunofluorescence staining for intranuclear Ki67 (Fig. 2E). Data were presented qualitatively on pictures and quantitatively on graphs. Percentage of the cells with positive Ki67 was calculated from 10 different pictures. Expression of Ki67 was significantly increased in 5‐AZA (<0.01) treated cells, representing an increased proliferative capacity in comparison to control culture (Fig. 2F).

The curve established for BrdU incorporation after 48, 96 and 168 hrs of culture displayed exponential character in both cultures. In 5‐AZA treated cells, we observed increased BrdU accumulation, but only on the seventh day, it was significantly higher in comparison to the control culture (*P* < 0.01) (Fig. 2G).

### The morphology and ultrastructure of ASCs treated with 5‐AZA supplemented culture medium

**Figure 3:**
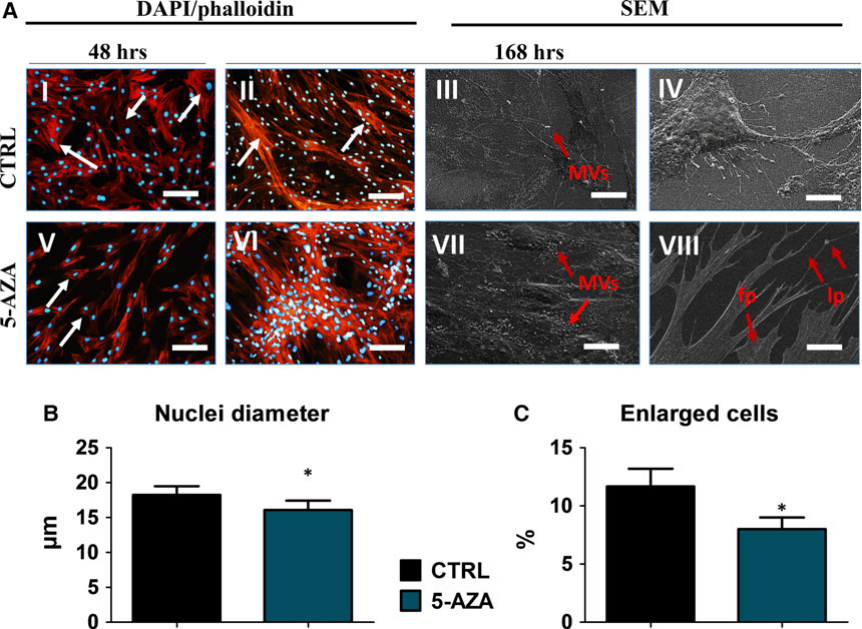
The effect of 5‐AZA treatment on hASCs morphology. Morphology of hASCs was evaluated after 48 and 168 hrs of the experiment using immunofluorescence and SEM imaging (**A**). Actin cytoskeleton was stained with Atto‐594 Phalloidin, whereas nuclei were counterstained with DAPI. After 48 of culture in control group, we observed small fraction of cells presenting spread‐out, flattened morphology and increased nuclei size, a typical features associated with ASCs senescence (pointed with white arrows on picture I). In contrast, cells cultured with 5‐AZA presented typical elongated, spindle‐shaped morphology. Interestingly, in this culture, small number of cells presented very small, round‐like morphology (indicated with arrows on picture V). After 168 hrs of culture cells reached high confluence in both groups, although after 5‐AZA treatment cells formed dense, multilayered aggregates and closely adhere to each other. Using SEM, we also evaluated production of MVs (III, VII). The production of MVs was more robust in 5‐AZA treated cells as we observed MVs not only on the edge of cell body but also around nucleus (VII). Those cells also developed a long structures known as filopodia and lamellipodia, which connected adjacent cells (VIII). To investigate whether 5‐AZA treatment influenced cells nuclei morphology, we evaluated mean nuclei diameter using SEM (**B**). Interestingly, we observed decreased nuclei diameter in 5‐AZA treated cells (*P* < 0.05). In addition, based on obtained photographs, percentage of enlarged cells was calculated (**C**). Magnification: immunofluorescece ×100, scale bars 250 μm; SEM III, VII ×2000, scale bars 30 μm; IV, VIII ×5000, scale bars: 10 μm. *P < 0.05. Abbreviations: MVs: membrane‐derived microvesicles; fp‐ filopodia; lp‐lamellipodia.

**Figure 4:**
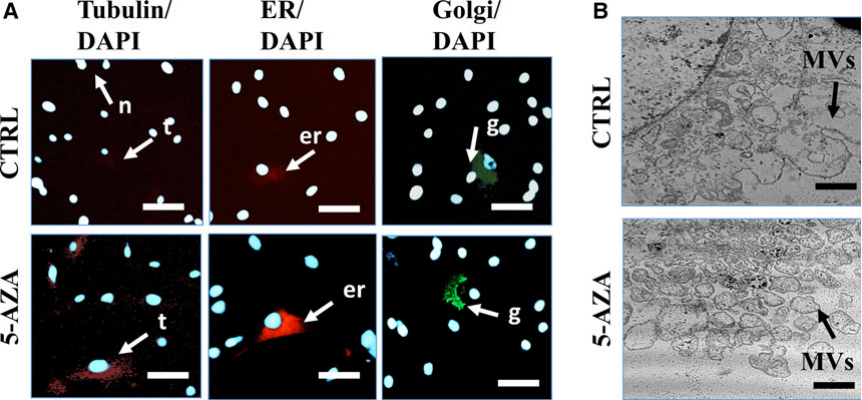
Assessment of hASCs organelles and microvesicles (MVs) production Cellular composition of hASCs (**A**), observed under epi‐fluorescent microscope. Morphological features are indicated with the following abbreviations: n: nucleus; t: tubulin; er: endoplasmic reticulum; g: Golgi apparatus. In control group, we observed decreased fluorescence intensity of tubulin and endoplasmic reticulum. Reduced tubulin signal may result from lower proliferation rate of cells from control group. Interestingly, both ER and Golgi intensity was also reduced in that group, impinging on decelerated metabolism rate. Magnification ×200, scale bars: 150 μm. Formation of MVs was visualized with TEM technique (**B**). A robust amount of MVs (membrane‐derived macrovesicles) was observed in 5‐AZA group (**B**). Magnification ×30.000, scale bars: 1 μm.

The morphological and ultrastructural characteristics of cells cultured with 5‐AZA was observed on seventh day of culture and compared to control culture. Analysis of cells using epi‐fluorescence microscopy revealed the characteristic features of ASCs morphology including elongated fibroblast‐like shape. In control culture, we observed small percentage of cells exhibited more flat, expended morphology with enlarged nucleus (indicated with white arrows on Fig. 3A I, II, V, VI). Interestingly in 5‐AZA, cultured cells displayed more spindle‐shaped, elongated morphology with no signs of apoptosis (no apoptotic bodies were observed). Moreover, cells treated with 5‐AZA were more concentrated, closely adhere to each other forming aggregates and multilayer after 168 hrs of culture. Analysis of cellular junctions formation showed that addition of 5‐AZA increased number of cytoskeleton projections, e.g. lamelli‐ and filipodia. In comparison, control group displayed the smallest‐scale cytonemes web connecting neighbouring cells. The number of plasma‐derived particles (microvesicles, MVs) was increased in 5‐AZA cells suggesting increased metabolic activity of those cells (indicated with red arrows and proper abbreviations on Fig. 3A III, IV, VII, VIII). Moreover, mean nuclei diameter was decreased in 5‐AZA cultures (Fig. 3B). Moreover, we observed that 5‐AZA treatment reduced the amount of enlarged cells in culture (Fig. 3C). Staining for organelles observed under fluorescence microscope, revealed more intense fluorescence signal for Tubulin in experimental group, which may partially explain higher proliferative activity of those cells. Similar, most intense endoplasmic reticulum (ER) signal was also observed in this group. Interestingly, abundance of Golgi Apparatus signal intensity was comparable in both investigated groups (Fig. 4A). In addition, we estimated production of MVs using TEM. The most robust production of those membrane‐particles was observed on the edge of ASCs treated with 5‐AZA (Fig. 4B).

### Treatment of ASC with 5‐AZA decreases apoptosis and senescence

**Figure 5:**
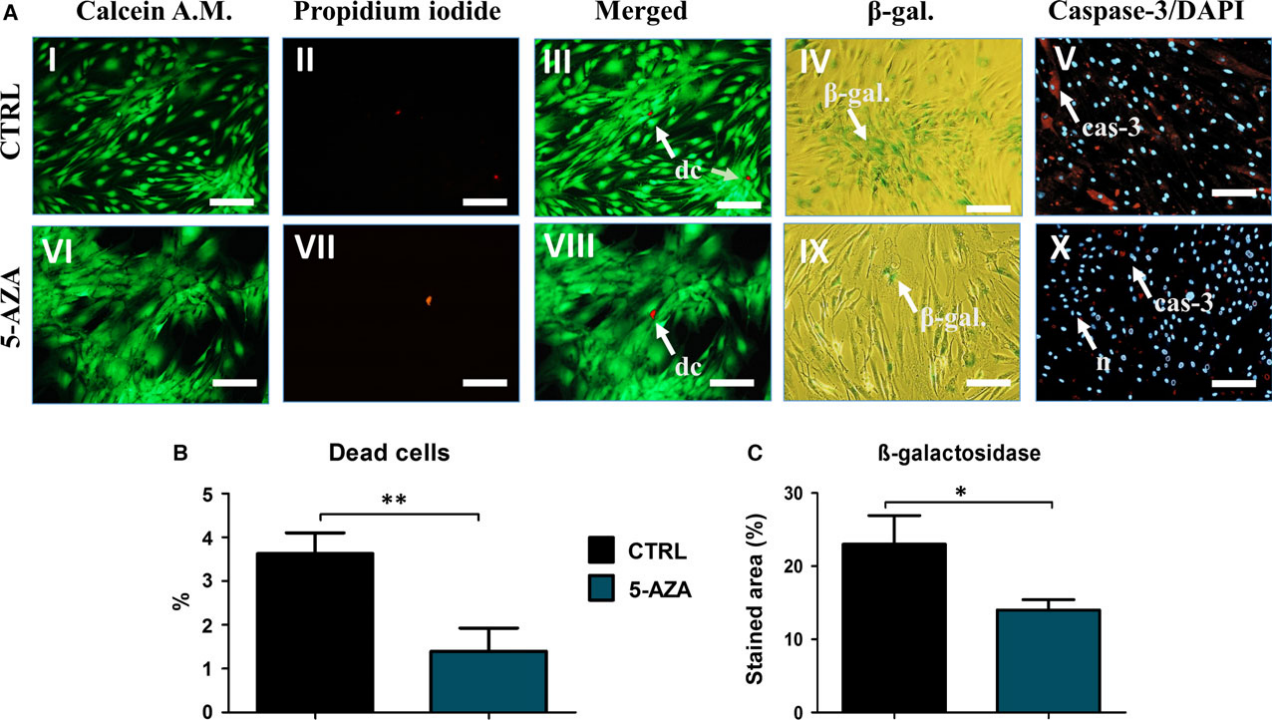
Evaluation of apoptosis and senescence in hASCs after 5‐AZA treatment. Representative pictures showing live cells (stained green with Calcein), dead cells (stained orange with Propidium Iodide) and merged (**A**). In addition, percentage of dead cells was calculated (**B**) showing less susceptibility for apoptosis in 5‐AZA culture cells (*P* < 0.01). Staining for senescence‐associated β‐galactosidase (blue) (IV and IX). Differences in the accumulation of β‐galactosidase were determined based on the percentage of stained area (**C**) showing decreased expression of β‐gal in cells cultured with 5‐AZA (*P* < 0.05). Moreover, immunofluorescence staining using anti‐caspase‐3 antibody, revealed higher expression of that protein in control group (V, X). Nuclei were counterstained with DAPI. Magnifications: ×100, scale bars 250 μm. Abbreviations: dc‐ dead cell, β‐gal‐β‐galactosidase, cas‐3‐ caspase 3. *P < 0.05, **P < 0.01.

To determine whether treatment with 5‐AZA can reduced apoptosis and senescence levels in ASCs we evaluated percentage of dead cells and stained cells for β‐galactosidase and caspase‐3 activity. Analysis of typical signs of cell death performed on seventh day of the experiment, using fluorescence and light microscopy. Live cells were stained green with Calcein AM, whereas dead cells were stained orange/red with Propidium Iodide (Fig. 5A). Obtained results showed that the percentage of dead cells was decreased in 5‐AZA treated cells (*P* < 0.01) (Fig. 5B). Senescence associated accumulation of β‐galactosidase (β‐gal) was stained blue on histology samples (Fig. 5A). Number of positive stained cells was decreased in 5‐AZA in comparison to control group. In addition, using computer software, we estimated percentage of stained area to provide quantitative data (Fig. 5C). Those results showed significantly reduced (*P* < 0.05) accumulation of β‐gal in 5‐AZA cultures. In turn, the expression of caspase‐3 staining in control group was higher when compared to ASCs cultured with 5‐AZA.

### 5‐AZA supplementation decreases oxidative stress in ASC

**Figure 6:**
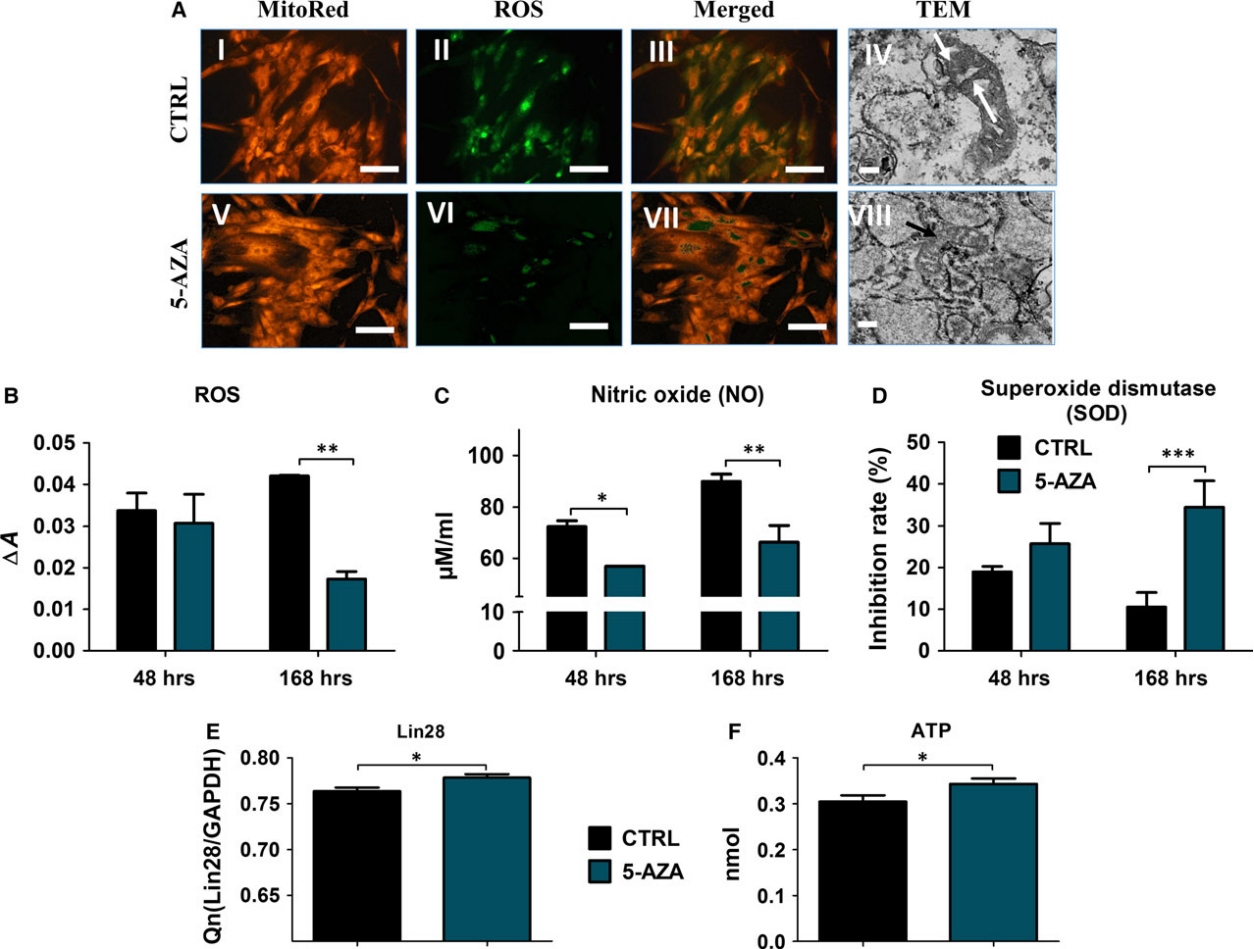
Oxidative stress factors in hASCs are reduced after 5‐AZA treatment. Images showing mitochondria stained with MitoRed dye (I, V) and accumulation of intracellular reactive oxygen species (ROS, II, VI). Merged pictures revealed that most of ROS was derived from mitochondria. To investigate whether the amount of ROS correlates with mitochondria structure, we performed their imaging with TEM ×30k, scale bars 1 μm. In control group, we observed that some of mitochondria had disarrayed membranes and formed vacuoles within its matrix (indicated with arrows, IV). In 5‐AZA treated cells although mitochondria were smaller in shape, they presented typical morphology, e.g. typical, bean‐shaped structure with intact membranes and numerous cristae (VIII). Magnification: fluorescence stainings ×100, scale bars: 250 μm, TEM: ×10k, scale bars: 2μ. Amount of ROS was also evaluated quantitatively with H2DCFDA after 48 and 168 hrs of culture (**B**). Moreover, we evaluated levels of nitric oxide and SOD. Both assays confirmed positive influence of 5‐AZA treatment on oxidative status of hASCs as it decreased nitric oxide production (**C**) simultaneously with increased antioxidative protection coming from SOD (**D**). Moreover, using RT‐PCR, we evaluated mean expression of Lin28 (**E**) which regulate the self‐renewal of stem cells and effects of its activation faded with age. After treatment with 5‐AZA, we observed increased expression of Lin28 transcript (*P* < 0.05) in hASCs. Moreover, we observed increased ATP production in 5‐AZA group (**F**). *P < 0.05, **P < 0.01, ***P < 0.001.

To determine whether 5‐AZA treatment can influence the level of oxidative stress factors, we evaluated the levels of ROS, nitric oxide and SOD after 48 and 168 hrs of culture. In addition, we imaged mitochondria using TEM. After fluorescence staining for ROS, we observed that ROS levels were decreased in 5‐AZA culture. When comparing ROS images with Mito Red staining, it is apparent that the most ROS is derived from mitochondria (Fig. 6A). Reduced ROS levels in experimental group suggest that 5‐AZA improved mitochondrial function in ASCs. To determine whether treating cells with 5‐AZA can affect mitochondria structure, we carried out TEM analysis. Some of mitochondria from control group displayed structure abnormalities including disarrayed cristae, membrane rapture and formation of vacuoles (indicated with arrows on Fig. 6A IV, VIII). On the contrary, mitochondria from cells treated with 5‐AZA presented typical bean‐shape structure with numerous cristae. Evaluated with spectrophotometer ROS levels, confirmed the staining results. After 48 hrs of culture ROS levels was comparable in both groups, while on seventh day of culture, it decreased in 5‐AZA treated ASCs (Fig. 6B). Nitric oxide levels were progressively increasing during culture, although it was significantly decreased in experimental group after both 48 (*P* < 0.05) and 168 (*P* < 0.01) hrs in comparison to control (Fig. 6C). The activity of SOD decreased during time in the control culture, while on the contrary, in 5‐AZA cultures increased during the culture period. Activity of SOD was higher in experimental group, but only on seventh day, it was statistically important (*P* < 0.001) (Fig. 6D). Moreover, the mRNA level of Lin28, was increased in ASCs after 5‐AZA treatment (Fig. 6E) (*P* < 0.05) in comparison to control group. Interestingly, ATP production was also increased in that group (Fig. 6F).

### Detection of BAX, p21, p53 and Bcl‐2 mRNA

**Figure 7:**
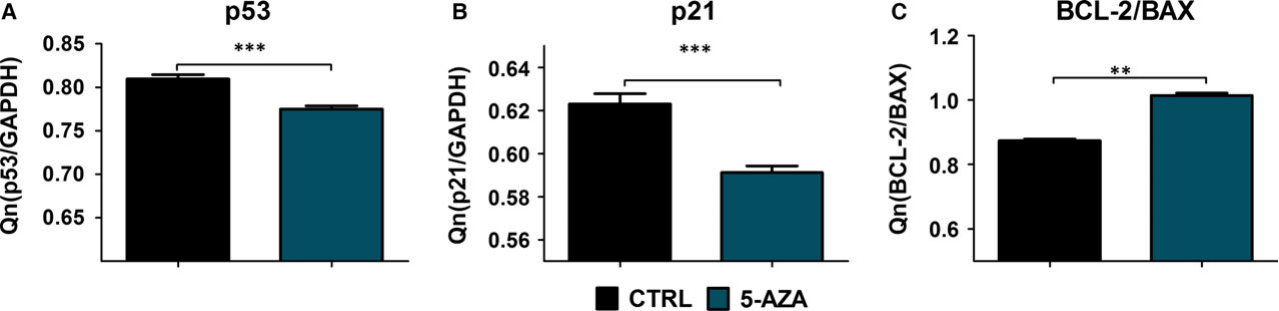
Reverse transcriptase–polymerase chain reaction (RT‐PCR) results. After 168 hrs of culture, total RNA was isolated from the cells and RT‐PCR analysis were performed. The expression of both p53 (**A**) and p21 (**B**) apoptosis‐related genes was significantly decreased in 5‐AZA cultures (*P* < 0.001). We also observed increased ratio of BCL‐2/BAX in 5‐AZA culture (**C**) (*P* < 0.01). *P < 0.05, **P < 0.01, ***P < 0.001.

Analysis of mitochondrial apoptosis related genes: BAX, p21 and also tumour suppressor p53 gene expression was analysed in ASCs form experimental and control cultures after 168 hrs of propagation. Quantitative analysis of transcripts revealed that expression of and p53 mRNA was significantly decreased in 5‐AZA cultures (*P* < 0.001) (Fig. 7A). Similarly, the level of p21 transcript was also decreased in experimental cultures (*P* < 0.05) (Fig. 7B). The ratio of Bcl‐2/BAX mRNA was increased (*P* < 0.01) after 5‐AZA treatment (Fig. 7C). GAPDH was used as an endogenous control.

### 5‐AZA treatment decreases DNA methylation status and increases TET gene expression

**Figure 8:**
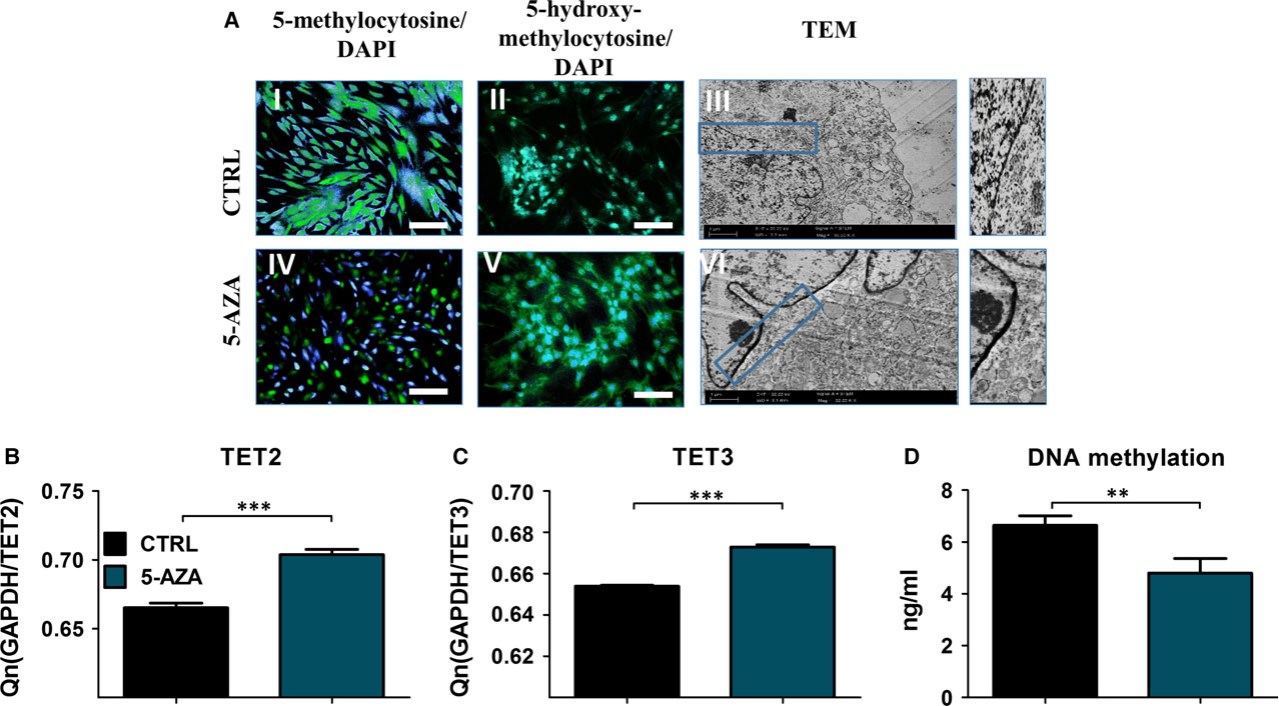
Distribution of 5‐methylocytosine (5‐MC), 5‐hydroxymethylocytosine (5‐hMC) and the TET mRNA levels. Genomic distribution of 5‐MC (I, IV) and 5‐hMC (II, V) was evaluated using immunofluorescence stainings to determine DNA methylation status. Nuclei were counterstained with DAPI. The 5‐ MC fraction was reduced after 5‐AZA treatment, while 5‐hMC fluorescence intensity was increased in this group in comparison to control. Using TEM imaging cells nuclei were visualized (III, VII). Higher magnification images of boxed regions shown on the right depict heterochromatin underneath the nuclear envelope. Interestingly, cells treated with 5‐AZA displayed broad heterochromatin architecture at the nuclear periphery. Both TET mRNA levels (**B,C**) were significantly increased (*P* < 0.001) after 5‐ AZA supplementation. Treating cells with 5‐AZA not only reduced DNA demethylation passively but also actively by inducing TET expression following increased 5‐hMC fractions. DNA methylation assessment using ELISA kit (**D**). Magnification ×100, scale bars 250 μm ***P < 0.001.

Genomic distribution of 5‐MC and 5‐hMC was evaluated by immunofluorescence staining after 168 hrs of culture to assess DNA methylation status. Nuclei were counterstained with DAPI. The distribution of investigated molecules was strongly affected by 5‐AZA (Fig. 8A). The 5‐MC fraction was more robust in control group with simultaneously decreased levels of 5‐hMC. In ASCs treated with 5‐AZA, 5‐MC fraction was decreased, which resulted in increased 5‐hMC positive fraction. We also determined relative gene expression of TET2 and TET3, which are responsible for active regulation of DNA methylation, after 168 hrs of propagation. GAPDH was used as an endogenous control. Both, TET2 and TET3 mRNA levels (Fig. 8B, C) were significantly increased after 5‐AZA treatment, which strongly correlates with the results of immunofluorescence methylation staining. Moreover, DNA methylation status was assessed quantitatively using ELISA test. We observed decreases in DNA methylation after 5‐AZA supplementation (Fig. 8D).

## Discussion

Currently, regenerative medicine as well as tissue engineering is searching for advance solutions, that can improve the regenerative process of different tissues or organ damages. Because of the capacity for self‐renew and capabilities to differentiate into multiple lineages, MSCs represent an important building block for cellular therapies. Adult MSCs of various origins has been involved in wide range of research, including ours, which proved their great therapeutic potential. However, during ageing of organism, MSCs are losing their ‘stemness’ character and thus, novel strategies that might rejuvenate their cytophysiological conditions *in vitro*, before clinical application, are strongly required 36. Here, we demonstrated that pre‐treatment of MSCs, isolated from elderly donors, using 5‐AZA might be a successful way to reverse their aged phenotype and increase therapeutic potential.

Ageing of stem cells is complex and yet, not fully understood process. The lifelong persistence of MSCs within the body, makes them susceptible for the accumulation of cellular damage which in turn leads to senescence, apoptosis and decreased regenerative potential 37. Those impairments cause blunted responsiveness for tissue injury, declining functional capacities and reduced effectiveness of cells replacement in aged organisms 37. Thus, it is crucial to understand the molecular pathways involved with age‐related impairment of MSCs. Here, we have explored common ageing phenotypes of hASCs including accumulation of ROS, antioxidative SOD protection, mitochondria dysfunction, epigenetic status and the influence of 5‐AZA treatment on their occurrence.

As previously showed, hASCs isolated from aged donors, although exhibit typical for MSCs morphology and phenotype (CD44^+^, CD73^+^, CD90^+^ and CD34^−^ CD45^−^) comparing to young patients, have suffered from lower expression of CD73 surface marker 15. Thus, bearing in mind that fact, in the current research, we investigated hASCs of aged donors that exhibited impairment of CD73 surface marker expression, for testing hypothesis if *in vitro* pre‐treatment using 5‐AZA might improve their viability and cellular activity.

Interestingly, obtained data indicates that 5‐AZA supplementation affects the effectiveness of ASC differentiation potential. In performed multipotency assay, we observed increased formation of extracellular matrix and proteoglycans during osteo‐ and chondrogenesis respectively. On the other hand, formation of lipid droplets during adipogenesis in 5‐AZA treated cells seems to be reduced. The effects of 5‐AZA on ASC differentiation needs further study to be elucidated in more details.

Since proliferation rate and viability of hASCs are considered as a one of the most important factors, that directly corresponds with regenerative process 38, we analysed proliferative activity (PA), PDT, viability, and potential for creating colonies originated from single cell (CFU‐fs) under the effect of 5‐AZA. We have found that hASCs isolated from aged patients, that were supplemented with 5‐AZA in comparison to not‐treated cells, however, still elderly patients cells, exhibited significantly elevated PA, shortens PDT and typical for MSCs spindle shape morphology. On the contrary, in control group we observed cells with spread‐out, flattened morphology and increased nuclei size, typical features associated with ASC senescence. What is more, we observed, that 5‐AZA improves CFU‐fs when compared to not stimulated cells. Obtained data stands in good agreement with Marinovic‐Kulisic *et al*., findings, that also observed pro‐proliferative effect of 5‐AZA in *in vitro* culture ofrat foetal epiglottis 39. Moreover, in the current research, we have found the highest percentage of Ki67^+^ as well as BrdU positive cells in hASCs treated with 5‐AZA comparing to not‐treated cells.

What is more, studies showed that not only the method of administration, but also timing of delivery and number of cells delivered are crucial in effectiveness of stem cells therapy 7. Treatment of cells with 5‐AZA enabled for extensive *in vitro* cell expansion, in short period of time, which seems to be crucial when taking into consideration above mentioned facts, costs of prolonged culture and replicative senescence. In addition, scanning and transmission electron microscope investigations revealed increased formation of membrane derived vesicles (MV's) in 5‐AZA treated cells. As it was shown by Ratajczak *et al*., 40 MVs play pivotal role in stem cell therapies because of their paracrine effects. It was proved that MVs riched in growth factors, cytokines, and RNA or microRNA migrate to injury site and initiate healing process of damaged organs. These observations from the regenerative process point of view seems to be fundamental, since elevated cellular activity together with increased paracrine activity of the cells treated with 5‐AZA may directly affects the course of tissue healing process *in vivo*.

That elevated cellular activity and viability of aged hASCs treated with 5‐AZA may be partially explain by the up‐regulation of *Lin28* mRNA. It was previously showed, that *Lin28* RNA binding protein, that expression is up‐regulated during embryogenesis, plays an fundamental role in development process, maintenance of pluripotency/multipotency as well as regulation of the cellular metabolism 41. Metabolism is now known to play pivotal role in dictating whether cell proliferate or not. Likewise, it was clearly demonstrated, that *Lin28* transcript promotes regeneration capacity in adult tissues by enhancing oxidative metabolism (glycolysis and oxidative phosphorylation) and promoting bioenergetic state characteristic of embryonic cells. It was shown that MSCs rely on glycolysis to remain quiescent state. Thus, switching MSCs metabolism towards oxidative phosphorylation may enhance differentiation process but on the other hand makes cells more susceptible for ROS damage 42, 43.

In turn, oxidative metabolism which might be negatively unsettled by the action of ROS and/or nitric oxide and impair MSCs cellular function 44. Increased amount of DNA methylation as well as accumulation of ROS during ageing is a well‐known phenomenon, observed in aged cells. As it was shown by Brandl *et al*. 45, aged MSCs are susceptible for oxidative stress damage, DNA strand breaks followed by increased p53 and p21 expression. Hence, application of 5‐AZA in *in vitro* culture of aged hASCs, that suffer from weaker activity of free radicals scavengers, like superoxide dismutase, might be an effective tool that abolishes the consequences of oxidative stress in aged hASCs. Here, we observed the lowest percentage of: (i) dead cells, (ii) senescence cells [β‐galactosidase positive cells] and (iii) caspase‐3 positive cells in this culture, where 5‐AZA was incorporated. Moreover, we have found, that aged hASCs accumulated robust amount of ROS within the mitochondria, simultaneously affecting their ultrastructure and function, what partially might explained impairment of proliferative activity of control, aged cells.

Previous findings of Stolzig *et al*. and others 46 clearly showed link between accumulation of oxidative stress factors and mitochondria impairment, especially in aged MSCs. Here, we confirmed this regularity, showing that 5‐AZA pre‐treatment of aged hASCs resulted in both: reduction in ROS and nitric oxide level with simultaneous increase in SOD activity in elderly donors derived hASCs. Furthermore, in accordance with Stolzig and others, elevated oxidative stress factors accumulation in aged donors stromal stem cells leads to activation of apoptotic action and finally through reducing their viability and mitochondria dysfunctions induce apoptosis. Our findings showed that those problems can be overcome by 5‐AZA treatment, as we observed increased activity of SOD, and decreased mRNA levels of pro‐apoptotic genes, i.e. p‐21 and p‐53. Simultaneously, we have found, the up‐regulation of BCL‐2/BAX ratio. Thus, taking into consideration above mentioned advantage of anti‐apoptotic genes expression over pro‐apoptotic, it might explain rejuvenated character of hASCs of elderly donors when compared with not‐treated cells.

In recent years, Lin28 emerged as a factor that define stemness in several tissue lineage, play important role in metabolism, somatic reprogramming and cancer. As it was also reported that Lin28 bound to and enhanced the translation of mRNAs for several metabolic enzymes, thereby oxidative phosphorylation, we decided to evaluate its expression under control and 5‐AZA conditions. We observed up‐regulation of Lin28 mRNA in 5‐AZA treated ASC. Increases of oxidative glucose metabolism were also confirmed by ATP assay as we observed increased production of ATP in 5‐AZA group. Thus, it is tempting to speculate that 5‐AZA anti‐ageing properties may results from increased Lin28 expression, which in turn activates mRNA translation and assembly of subunits of the mitochondrial complex and switches in glucose metabolism.

Because of the great therapeutic potential of hASCs, wide range of studies has been performed to investigate stem cell metabolism, including functional modification of gene expression and epigenetic modulations. Still, the data regarding the effects of epigenetic regulators remain controversial. Study conducted by Rui *et al*. revealed that epigenetic alternations of MSCs could be an attractive way to control their differentiation 47. Similarly, Qian *et al*. showed that 5‐AZA increased MSCs cardiomyogenesis 48.

Moreover, studies conducted by Yan *et al*., revealed that 5‐AZA, which inhibits the activity of DNA methyltransferases (DNMTs), promoting DNA demethylation in aged hASCs simultaneously improves their osteogenic differentiation potential. 5‐AZA has been demonstrated to be easily incorporated into DNA, methylate specific genes and finally induces TET family genes expressions that convert nuclear 5‐methylcyt‐osine (5 mC) to 5 hmC 49. Here, we have observed, that 5‐AZA pre‐treatment in aged donors of hASCs abundantly transferred 5‐methylcyt‐osine (5 mC) to 5 hmC. In addition, immunofluorescence data were confirmed quantitatively, as we observed decreased DNA methylation status after 5‐AZA supplementation. Moreover, we noticed the higher expression of TET2/TET3 transcripts on mRNA level that taking part in the process of 5mC to 5hmC transformation. Targeting DNA methylation for epigenetic therapy involves application of hypomethylation agents in the clinic to restore the proper methylation status of cells. Our results indicates and confirmed demethylative character of 5‐AZA in elderly donors hASCs, and thus might become an essential factor in the process of hASCs rejuvenation.

Taken together, MSCs contribute to peripheral tissue repair *in vivo* and form a cell source for regenerative medicine. Their regenerative potential is impaired by cellular senescence and highly depends on donor age. Accumulation of oxidative stress and increased methylation status of DNA are well described phenomenons, which lead to MSCs senescence. Our results indicates that, treating hASCs with 5‐AZA can be justified therapeutic intervention, that can slow‐down and even reverse aged‐ related degenerative changes in those cells.

## Conflict of interests

The authors confirm that there are no conflicts of interest.
